# Supplementary material for: Early and Late Transcriptomic and Metabolomic Responses of Rhododendron ‘Xiaotaohong’ Petals to Infection with Alternaria sp
Source: Int J Mol Sci. 2023 Aug 11;24(16):12695. doi: 10.3390/ijms241612695 (PMC10454523; doi:10.3390/ijms241612695)
Supplement: Supplementary file 1 [file ijms-24-12695-s001.zip › ijms-2524285 Supplementary Figures.pdf]

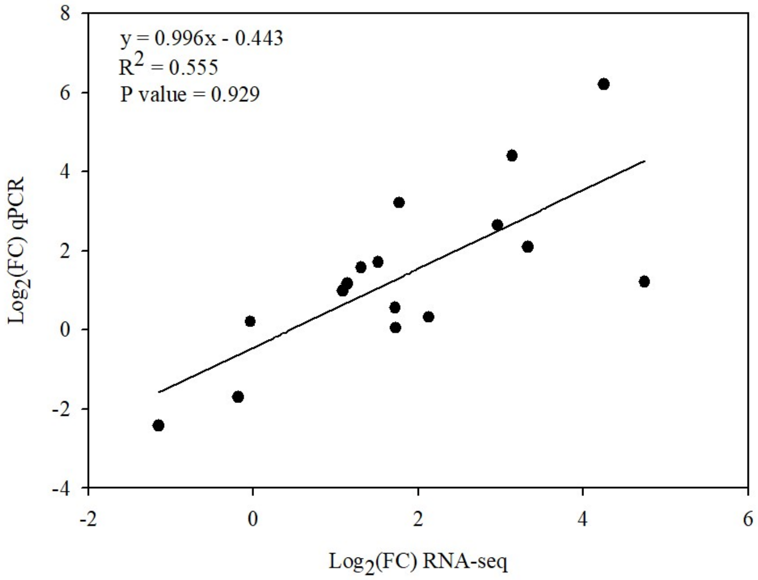

Figure S1. Validation of RNA-Seq results using qRT-PCR. Significantly positive correlation of 8 genes between RNA-Seq and qRT-PCR (R^2 = 0.555). Fold change (FC) represent the expression levels of each gene in infection petals relative to its respective mock in 12hpi and 144hpi. The relative expression levels of eight genes used in this study were detected using qRT-PCR in mock and infection petals in 12hpi and 144hpi. The relative expression levels of the selected genes normalized to the expression level of *EF1α* gene were calculated from cycle threshold values using the 2-ΔΔCT method. The correlation between $\log_2$(FC)RNA-seq and $\log_2$(FC)qPCR were used by linear polynomial.

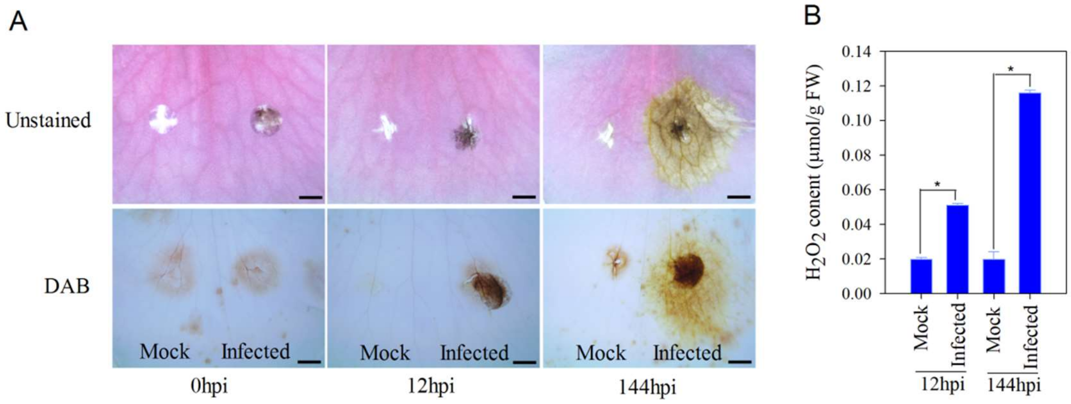

Figure S2. Hydrogen peroxide (H_2O_2) distribution and content analysis. **(A)** DAB staining in mock and infection petals in 12hpi and 144hpi showed H_2O_2 distribution around blight spots, bars = 2 mm. **(B)** H_2O_2 content in mock and infection petals in 12hpi and 144hpi. Asterisks represent significantly differences between infection and mock.

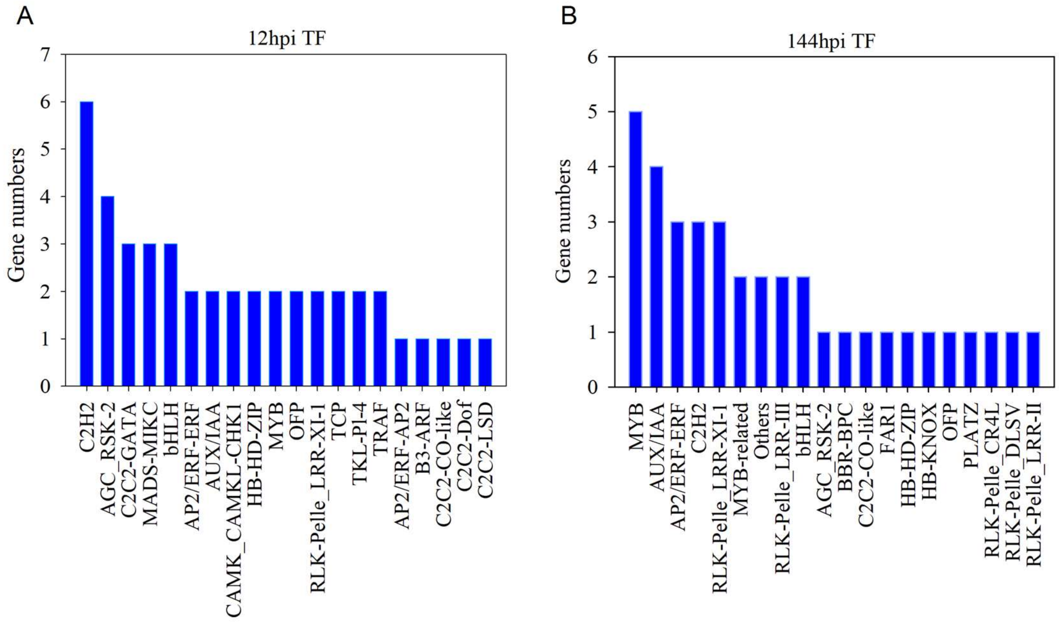

Figure S3. The predicting transcription factors (TFs) encoded by DEGs in 12hpi petals. **(A)** The TFs encoded by down-regulated DEGs in 12hpi; **(B)** The TFs encoded by down-regulated DEGs in 144hpi.

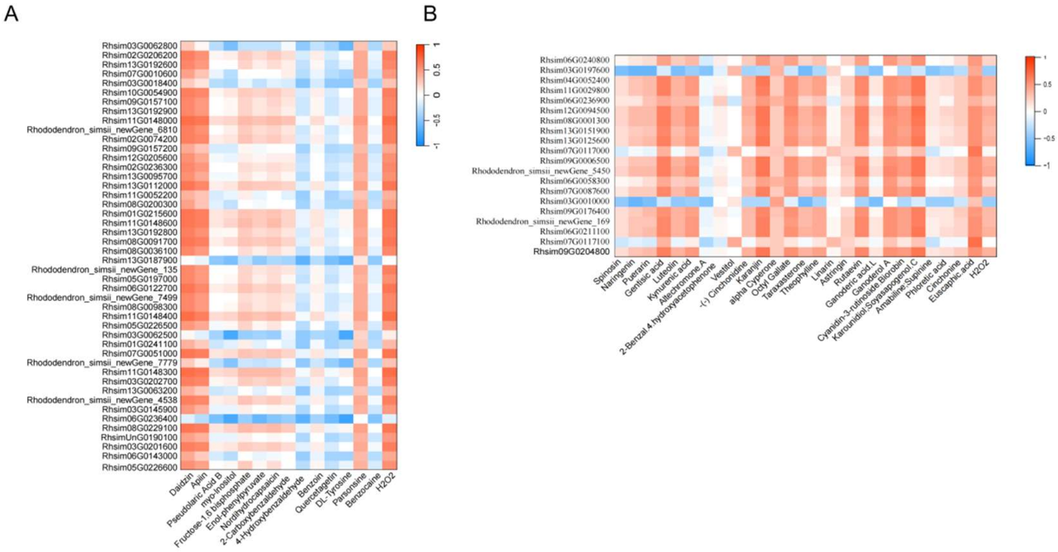

Figure S4. Correlation analysis of up-regulated DEGs in the plant-pathogen interaction pathway and accumulated metabolites. (**A**) the infected petals at 12hpi. (**B**) the infected petals at 144hpi ($P < 0.05$).
